# Completing Single-Cell DNA Methylome Profiles *via* Transfer Learning Together With KL-Divergence

**DOI:** 10.3389/fgene.2022.910439

**Published:** 2022-07-22

**Authors:** Sanjeeva Dodlapati, Zongliang Jiang, Jiangwen Sun

**Affiliations:** ^1^ Department of Computer Science, Old Dominion University, Norfolk, VA, United States; ^2^ School of Animal Sciences, AgCenter, Louisiana State University, Baton Rouge, LA, United States

**Keywords:** DNA methylation, single cell WGBS, embryo methylome, methylation imputation, transfer learning, KL divergence

## Abstract

The high level of sparsity in methylome profiles obtained using whole-genome bisulfite sequencing in the case of low biological material amount limits its value in the study of systems in which large samples are difficult to assemble, such as mammalian preimplantation embryonic development. The recently developed computational methods for addressing the sparsity by imputing missing have their limits when the required minimum data coverage or profiles of the same tissue in other modalities are not available. In this study, we explored the use of transfer learning together with Kullback-Leibler (KL) divergence to train predictive models for completing methylome profiles with very low coverage (below 2%). Transfer learning was used to leverage less sparse profiles that are typically available for different tissues for the same species, while KL divergence was employed to maximize the usage of information carried in the input data. A deep neural network was adopted to extract both DNA sequence and local methylation patterns for imputation. Our study of training models for completing methylome profiles of bovine oocytes and early embryos demonstrates the effectiveness of transfer learning and KL divergence, with individual increase of 29.98 and 29.43%, respectively, in prediction performance and 38.70% increase when the two were used together. The drastically increased data coverage (43.80–73.6%) after imputation powers downstream analyses involving methylomes that cannot be effectively done using the very low coverage profiles (0.06–1.47%) before imputation.

## 1 Introduction

DNA methylation, a process of adding a methyl group to the fifth carbon of cytosines, is ubiquitous in genome of all kingdoms of life from bacteria to eukaryotes (Zemach et al., 2010). Although there exist methylated cytosines in other contexts, methylation in the context of CpG dinucleotides (i.e., a cytosine nucleotide being immediately followed by a guanine nucleotide along the 5’ → 3’ direction of a sequence) is the most common form (Feng et al., 2010) and is the subject of this study. DNA methylation plays critical roles in the regulation of both genome stability and gene expression (Greenberg and Bourc’his, 2019), involved in many important biological processes such as embryonic development (Zhu et al., 2018; Duan et al., 2019), X-chromosome inactivation (Grant et al., 1992), genomic imprinting (Proudhon et al., 2012), and aging (Xiao et al., 2019). Alterations in the usual methylation patterns may lead to disruption of normal cellular functions and disease conditions. Disrupted DNA methylation has been linked to several diseases such as cancer (Ko et al., 2010; Russler-Germain et al., 2014), immunological disorders (Rajshekar et al., 2018), and neurological disorders (Sun et al., 2014; Kernohan et al., 2016).

Due to its importance, obtaining DNA methylome profiles for varying biological systems has attracted considerable attentions (Abascal et al., 2020). Several techniques have been developed for profiling DNA methylation genome-wide, including methylated DNA immunoprecipitation sequencing (MeDIP-Seq) (Taiwo et al., 2012), whole-genome bisulfite sequencing (WGBS) (Clark et al., 2017), reduced representation bisulfite sequencing (RRBS) (Gu et al., 2011), and nanopore sequencing Clarke et al. (2009) followed by methylation detection. Since MeDIP-Seq relies on a methyl-cytosine antibody to pull down methylated DNA fragments followed by sequencing, the obtained profiles, even though genome-wide, are in low resolution (100–300 bp) and biased, with substantial underrepresentation of CpG poor regions (Rauluseviciute et al., 2019), limiting its application in biological studies. Nanopore sequencing, one of the emerging third-generation sequencing techniques, is capable of producing reads of much longer length (in tens to hundreds of thousands bases) compared to their short-read sequencing counterparts. Several computational approaches have been developed to predict DNA methylation from nanopore sequencing reads (Yuen et al., 2021). However, due to limited accuracy in both sequencing and subsequent methylation prediction (Liu et al., 2021), nanopore sequencing has yet become a widely used approach for methylome profiling.

Both RRBS and WGBS are based on bisulfite conversion and capable of producing methylome profiles at single-base pair resolution. Without the bias of RRBS for CpG dense regions, WGBS is currently the most popularly used methylome profiling technology and has been used to obtain profiles for a wide range of tissues in varying organisms (Roadmap Epigenomics Consortium et al., 2015; Abascal et al., 2020). However, to obtain a profile with high data coverage rate (defined as the proportion of CpG sites with profiled methylation state out of the total in the entire genome) using WGBS, large amount of genetic input coupled with high sequencing depth is required. Single-cell WGBS is well known for its very low coverage rate. When excluding CpG sites with low amount (below 5) of overlapping reads, the data coverage rate in single-cell methylomes can get down to just a little over 1% (Zhu et al., 2018) or even well below 1% (Smallwood et al., 2014). In applications, such as the study of mammalian preimplantation of embryos where genetic material is precious, the coverage rate can go extremely low after rigors data cleaning (see Materials and Methods), only 0.06–1.47% (all but one below 0.3%) in a recent study of bovine embryonic development (Duan et al., 2019). Sparsity in methylome profiles hinders the downstream analyses, limiting their value in efforts to understand the dynamics and regulation of biological processes.

To address the sparsity in DNA methylome profiles, many computational approaches have been developed in the past to impute missing data by training machine learning models to predict methylation state. With the advancement of technologies for assessing DNA methylation, the computational approaches have shifted from predicting the overall methylation level of a DNA fragment such as a CpG island (Bock et al., 2006) to the methylation state of individual CpG sites (Zhang et al., 2015). Varying types of data have been explored to use as input to predict DNA methylation, including a variety of DNA sequence patterns, methylation state of neighboring CpGs, profiles of other functional genomic events such as histone modifications in the same sample, and epigenetic profiles of other related samples. By leveraging a diverse of genomic profiles, several methods achieved very high prediction accuracy. For example, BoostMe (Zou et al., 2018) obtained an accuracy that is above 0.96 with using profiles of 7 histone markers, predicted binding sites of 608 transcriptional factors, predictions for 13 chromatin states, and chromatin accessibility profiles by assay for transposase-accessible chromatin with sequencing (ATAC-Seq). However, these methods have limited usage in the study of biological systems for which a wealth of additional data are not available.

Earlier methods used hand-crafted features derived from DNA sequence, which is limited by the understanding of the biology at the time and leads to suboptimal results. With seeing the successful applications of deep neural networks (DNNs) in many other domains, especially computer vision (Krizhevsky et al., 2012) and natural language processing (Otter et al., 2021), several recent studies have attempted to use DNNs to learn unbiased DNA sequence and/or local methylation patterns (Sharma et al., 2017; Zeng and Gifford, 2017; De Waele et al., 2022). Even though with success to some extent, these methods are limited by the availability of sufficient amount of data for training the DNNs. Transfer learning performs well in various low amount data scenarios by transferring knowledge learned on a large dataset that is different but related to the target learning problem (Zhuang et al., 2020). Several methods utilizing transfer learning have been developed recently in genomic data contexts where limited data are available, such as in the prediction of cancer survival using gene expression data (López-García et al., 2020), molecular cancer classification (Sevakula et al., 2018), denoising single-cell transcriptomics data (Wang et al., 2019), and imputing missing data in gene expression profiling with the input from DNA methylation profiles (Zhou X. et al., 2020). To the best of our knowledge, transfer learning has not been explored to leverage profiles with higher coverage rate in training predictive models for much sparser methylation profiles.

Due to allelic methylation, intercellular variability, or clusters of interspersed methylated and unmethylated CpGs within each cell, the intermediate DNA methylation (represented by a value in between 0 and 1) is widespread in the genome (Elliott et al., 2015). It has been indicated that intermediate methylation states may be functional and are dynamically regulated (Stadler et al., 2011). Moreover, large amount of methylome profiles that are available in public repositories were obtained by averaging across a group of cells that may be heterogeneous. Therefore, the variation in (intermediate) methylation level among CpG sites is indicative of difference in the context that regulates their methylation, such as the surrounding DNA sequence. However, when training models for predicting methylation by gradient descent to optimize a concrete objective, previous works chose to convert methylation level to the binary on or off state followed by employing a binary classification loss function such as logistic loss (Painsky and Wornell, 2018). Such an binary conversion results in loss of information and may lead to suboptimally trained models. Technically, to avoid binary conversion, the learning problem can be modeled as a general regression problem, where mean squared error (MSE) can be applied as the loss function with or without a final sigmoid mapping to ensure the model prediction within [0,1]. However, sigmoid mapping drives model outputs towards either 0 or 1, likely leading to suboptimal models; and, if without the sigmoid mapping, the model can output values beyond [0,1], making the prediction difficult to interpret. Kullback-Leibler (KL) divergence (Kullback and Leibler, 1951) that measures the difference between two distributions can be a better choice as a loss function for training classifiers without binary conversion, but so far has not been exploited in DNA methylation prediction.

Here in this article, we report the results from the exploration of using transfer learning together with KL divergence to train DNNs for completing DNA methylome profiles with extremely low coverage rate by leveraging those with higher coverage. We employed a hybrid network architecture adapted from DeepGpG (Sharma et al., 2017), a mixture of convolutional neural network (CNN) and recurrent neural network (RNN). The CNN learns predictive DNA sequence patterns and the RNN exploits known methylation state of neighboring CpGs in the target profile to complete and across others. To obtain pretrained network components (i.e., subnetworks), we used bovine methylome profiles of varying somatic tissues downloaded from NCBI GEO under accession numbers: GSE106538 and GSE147087. The majority of these profiles have a data coverage rate greater than 5% after cleaning (see Materials and Methods). The pretrained subnetworks were then transferred for the training of models to complete profiles of bovine oocytes and early embryos, which was also obtained from NCBI GEO (GSE121758). All of these profiles except one have a data coverage rate below 0.3%. The results from our empirical study indicate both model transferring and the use of KL divergence help to improve the performance of trained DNNs. Specifically, on average, there is about 22.45% increase in the performance measured in F1 score with model transferring and about 29.43% increase when using KL divergence. The use of both leads to even higher increase (about 38.70%), which suggests that the contributions of the two are in different nature and can be combined. The subsequent imputation using the trained DNNs increased the data coverage rate to 43.80–73.65% from the initial 0.06–1.47% for profiles of bovine oocytes and early embryos. The expanded data enable the methylation quantification for substantially more genomic features, such as genome bins, promoters, and CpG islands (CGIs). This could in turn lead to more insights into the dynamics in methylomes of bovine oocytes and early embryos across different stages and the understanding of roles of DNA methylation in regulating varying biological functions.

## 2 Background and Related Work

There has been a wealth of research work on building computational models to predict DNA methylation since the pioneer work in 2005 that trained a support vector machine (SVM) for predicting methylation level of short DNA fragments (Bhasin et al., 2005). Limited by the lack of technologies for obtaining data in high resolution, the majority of earlier works focused on the prediction of methylation level of CpG islands, genomic regions that are rich of CpG sites (Bock et al., 2006; Das et al., 2006; Fang et al., 2006; Fan et al., 2008; Zheng et al., 2013). The input used in the prediction comprised varying sequence features derived from DNA fragment in initial works (Bock et al., 2006; Das et al., 2006; Fang et al., 2006) and later was expanded to consider chromatin state of histone modifications including both methylation (Fan et al., 2008) and acetylation (Zheng et al., 2013). The used DNA sequence features typically included characteristics of CpG islands such as G + C content and CpG ratio, evolutionary conservation, count of k-mers, and occurrence of (predicted) transcription factor binding sites and repetitive elements such as AluY. Due to the small size of available data, machine learning algorithms that work well on small datasets were typically employed, including SVM, linear discriminant analysis, and logistic regression. Among them, SVM was used most often and frequently led to models that had the best performance. Even though these earlier approaches predict accurately the methylation level of CpG islands, they offer limited view of the involvement of DNA methylation in biological functions, because many functional elements such as enhancers are frequently located outside of CpG islands (Li et al., 2021).

Thanks to the rapid advancement of high-throughput sequencing technologies, profiling genome-wide DNA methylation at single base resolution has become possible and with increasingly low cost. Large numbers of genome-wide DNA methylation profiles of a wide range of tissues and cell lines for varying organisms have been deposited in public accessible data repositories such as ENCODE (Dunham et al., 2012), Roadmap (Roadmap Epigenomics Consortium et al., 2015), and NCBI GEO. The availability of these high-resolution genome-wide profiles enables the training of machine learning models that predict DNA methylation at individual CpG sites, which has become the primary target of recently developed approaches for methylation prediction.

Depending on the type of input, the methods for methylation prediction at individual base resolution can be generally classified into three categories. The first category includes methods that predict from coarse profiles obtained with MeDIP-Seq and Methylation-sensitive Restriction Enzyme sequencing (MRE-Seq) (Stevens et al., 2013), or methylation state of neighboring CpGs and methylation profile of other (related) samples (Ma et al., 2014; Kapourani and Sanguinetti, 2019; Yu et al., 2020; Tang et al., 2021), or additionally with the help from profiles for other epigenetic markers, such as histone modifications (Ernst and Kellis, 2015; Zou et al., 2018). Due to the availability of large amount of data for training, the most popularly used machine learning algorithm by these approaches is ensemble trees, either random forest or gradient boosting machines. To make accurate prediction using these approaches, either relative high data coverage or the availability of profiles of many other epigenetic markers is needed. The second category consists of methods that employ only the sequence features derived from the DNA fragment centered at the target CpG site to predict for. These methods vary mainly in the length of input DNA fragment and ways of deriving sequence features that include simply treating the input sequence as structured data (in other words each position is taken as an individual input variable) (Kim et al., 2008), counting of k-mers (Lu et al., 2010; Zhou et al., 2012), and using a CNN (Zeng and Gifford, 2017). The models obtained using these methods generally make less accurate prediction than those from the first category. The third category consists of methods that leverage both sequence features and functional chromatin states to varying extent, including methylation state of neighboring CpGs. There are methods relying on hand-crafted DNA sequence features similar to those approaches developed for predicting methylation level of CpG islands (Zhang et al., 2015; Jiang et al., 2019), but with the majority employing DNNs to derive features that are unbiased (Wang et al., 2016; Sharma et al., 2017; Fu et al., 2019; Levy-Jurgenson et al., 2019; De Waele et al., 2022). Notably, methods using DNNs generally perform better than those not when there is no additional input beyond the methylation profile of the target sample (Sharma et al., 2017; De Waele et al., 2022). However, it is well known that training DNNs is difficult, requiring large amount of training data. Therefore, the success in the application of existing DNN-based methods is limited when methylation profiles are extremely sparse.

Transfer learning is able to mitigate data scarcity problems of target domain by learning model priors on larger data in a source domain related to the target domain but with different data distribution. It has been shown with effectiveness in the learning for various low data scenarios (Zhuang et al., 2020). Different transferring strategies have been developed, among which instance-based, mapping-based, network-based, and adversarial-based are more prominent approaches (Tan et al., 2018). It has been reported that logistic loss is not effective in learning features for transferring (Islam et al., 2021), since it results in hard class separation and hence leads to less adaptability of the source model while transfer it to the target domain. This problem is acute when very few examples for training are available in target domain. Recently, transfer learning has been applied to impute incomplete RNA-sequencing data by transferring features learned during predicting DNA methylation (Zhou X. et al., 2020). To the best of our knowledge, transfer learning has not been explored to train DNN-based models for predicting DNA methylation to impute sparse methylomes.

## 3 Materials and Methods

To enhance downstream analyses, such as gene expression regulation, we train DNNs to impute missing methylation data in methylome profiles with the consideration of both DNA sequence patterns and methylation state of neighboring CpG sites. It is known that well-performing DNNs require large data in their training. However, methylome profiles of oocytes and mammalian preimplantation embryos are typically very sparse due to low amount of genetic material available for sequencing, limiting the amount of data for training DNNs. As a result, it is a challenging problem to obtain trained DNNs that make accurate predictions for missing CpGs in these profiles. To improve prediction accuracy of DNNs, we 1) employ the Kullback-Leibler (KL) divergence as the loss function in training to maximize usage of information carried in the data and 2) leverage transfer learning to make use of the much denser methylation profiles that are available for other tissues. Specifically, in this study, we trained DNNs for imputing missing CpG sites in methylome profiles of oocytes and preimplantation embryos of bovine.

### 3.1 Datasets

The methylome profiles of bovine oocytes and preimplantation embyros were obtained by downloading from NCBI GEO repository with accession number GSE121758. These profiles were produced via WGBS in a recent study of mythylome dynamics of oocytes and *in vivo* early embryos of bovine (Duan et al., 2019). More specifically, there are profiles for three types of oocytes, including two *in vivo* at different developmental stages, that is, germinal vesicle (GV) oocyte and metaphase II (MII) oocyte, and one *in vitro* MII oocyte. The dataset includes profiles for *in vivo* embryos at four different developmental stages: 2-cell, 4-cell, 8-cell, and 16-cell. The data coverage rate, that is, the proportion of CpG sites in the whole genome with known state in a methylation profile, is very low among these profiles, ranging from 0.06% for in vio MII oocyte to 1.47% for 16-cell embryo with all but one below 0.3% (Table 1).

**TABLE 1:** Summary of used bovine WGBS profiles.

Profile	Tissue	Accession number	Source	Breed	Data coverage rate (%)	Methylation rate (%)
Sperm	Sperm	GSE106538	Gamete	Holstein	21.34	74.11
MamGl	Mammary gland	GSE106538	Somatic	Holstein	12.6	73.59
PreCor	Prefrontal cortex	GSE106538	Somatic	Holstein	9.94	84.16
WBC1	White blood cell	GSE106538	Somatic	Holstein	15.38	81.22
WBC2	White blood cell	GSE147087	Somatic	Holstein	13.04	86.53
Adip1	Adipose	GSE147087	Somatic	Holstein	7.98	82.5
Adip2	Adipose	GSE147087	Somatic	Hereford	1.09	94.04
Muscle	Muscle	GSE147087	Somatic	Holstein	7.99	79.1
Heart1	Heart	GSE147087	Somatic	Holstein	1.32	80.44
Heart2	Heart	GSE147087	Somatic	Hereford	0.85	92.47
Lung	Lung	GSE147087	Somatic	Holstein	7.09	78.53
Spleen	Spleen	GSE147087	Somatic	Holstein	11.5	83.85
Liver1	Liver	GSE147087	Somatic	Holstein	5.46	83.27
Liver2	Liver	GSE147087	Somatic	Hereford	0.88	88.6
Ileum	Ileum	GSE147087	Somatic	Holstein	8.69	79.6
Rumen	Rumen	GSE147087	Somatic	Holstein	5.18	59.92
Jejun	Jejun	GSE147087	Somatic	Hereford	1.17	81.84
Kidn1	Kidney	GSE147087	Somatic	Hereford	1.28	88.31
Kidn2	Kidney	GSE147087	Somatic	Holstein	5.69	84.03
Uterus	Uterus	GSE147087	Somatic	Holstein	6.09	84.91
Ovary	Ovary	GSE147087	Somatic	Holstein	11.1	73.01
Placenta	Placenta	GSE147087	Somatic	Hereford	0.98	40.81
GVO	GV Oocyte	GSE121758	Gamete	Holstein	0.16	4.3
MIIO1	MII Oocyte	GSE121758	Gamete	Holstein	0.06	8.39
MIIO2	MII Oocyte^a^	GSE121758	Gamete	Holstein	0.13	5.05
2-Cell	Embryo	GSE121758	Embryo	Holstein	0.26	2.41
4-Cell	Embryo	GSE121758	Embryo	Holstein	0.21	3.32
8-Cell	Embryo	GSE121758	Embryo	Holstein	0.18	1.97
16-Cell	Embryo	GSE121758	Embryo	Holstein	1.47	5.94

a In vitro oocyte. All other oocytes and embryos are *in vivo*.

To enhance the training for oocytes and early embryos, we identified two bovine WGBS datasets in the NCBI GEO repository with accession numbers: GSE106538 and GSE147087, respectively. Both datasets provide methylome profiles for somatic tissues for which large amount of genetic materials are available for sequencing. Specifically, GSE106538 provides profiles for sperm in addition to three different somatic tissues of Holstein cattle: mammary gland, prefrontal cortex, and white blood cell (Zhou et al., 2018), while GSE147087 provides methylome profiles with varying availability for cattle of two different breeds: Holstein and Hereford for a total of 14 tissues, including lung, heart, spleen, kidney, liver, rumen, jejun, ileum, ovary, uterus, placenta, white blood cell, muscle, and adipose (Zhou Y. et al., 2020). Profiles included in GSE106538 have high data coverage rate, ranging from 9.94% for prefrontal cortex to 21.34% for sperm (Table 1). Compared to these profiles, the data coverage rate of profiles from GSE147087 is much lower, ranging from 0.95 to 13.04% with the majority above 5% (Table 1), which is still significantly higher than that in profiles of oocytes and early embryos.

To prepare data for network training and subsequent imputation, downloaded datasets underwent a sequence of preprocessing steps. First, the profiles that are replicates of the same tissue were merged within the same data source. Following the consolidation, we excluded CpGs from a profile that have limited support for their profiled methylation state, that is, with a number of overlapping sequencing reads no greater than 3. DNA methylation is known to be stable during replication and remains symmetric, meaning that the copy of the cytosine on one strand at a CpG site is expected to have the same methylation state as the copy on the other strand (Vandiver et al., 2015; Petryk et al., 2020). In other words, hemi-methylated (unsymmetrical) CpG sites are rare and the existence of such CpGs is high likely due to errors in the methylation profiling. To ensure high data quality and avoid causing confusion during the network training by ambiguous labeling, we excluded from all profiles the hemi-methylated CpG sites and those with data for only one strand. The data coverage rate in each profile after going through all the preprocessing steps, together with the methylation rate calculated using the remaining CpG sites, is provided in Table 1.

### 3.2 Network Architecture

**FIGURE 1:**
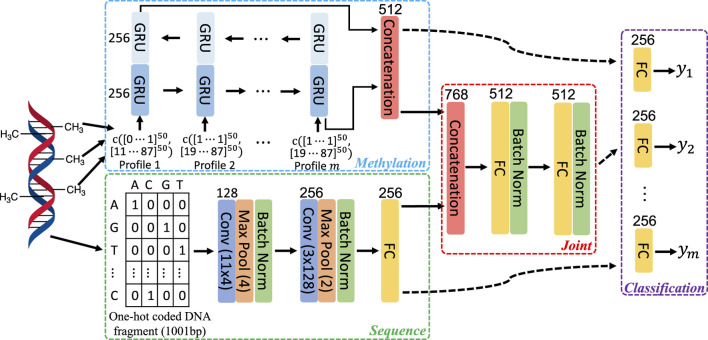
Architecture of network components in our study. Each colored bar represents a layer of operation in the network as indicated by the enclosed description. The numbers on the top or the side of bars specify the number of filters or hidden units in the corresponding layers. Activation functions following convolutional or fully connected layers and that in GRU are not shown. The former uses rectified linear function, while in the latter, hyperbolic tangent function is employed. ${\left[d_{1}\cdots d_{50}\right]}^{50}$ represents a numerical vector of size of 50 and *c* (**v**_1_, **v**_2_) denotes the concatenation of two vectors: **v**_1_ and **v**_2_. *y*_*i*_’s represent methylation state of the single CpG to predict in multiple profiles. When transferring trained models from source to target, components of Sequence, Methylation, Joint, or their combinations are transferred. Components that are not transferred are trained from scratch (i.e., random initialization). Conv, convolution; Max Pool, max pooling; Batch Norm, batch normalization; FC, fully connected.

To leverage both DNA sequence patterns and correlation in methylation state among neighboring CpGs, we employed networks with the architecture adapted from one that has been utilized for predicting DNA methylation in human and mouse genome (Angermueller et al., 2017). As illustrated in Figure 1, three feature learning subnetworks: Sequence, Methylation, and Joint were used to extract features from the input. Specifically, the Sequence subnetwork learns DNA sequence patterns that are predictive to methylation; the Methylation subnetwork learns correlation in the methylation state among neighboring CpGs; and the Joint subnetwork fuses the features extracted by the Sequence and Methylation subnetworks.

To learn sequence features, the Sequence subnetwork takes in one-hot coded DNA fragment of 1,001 base pair (bp) long, centered at the CpG to predict for and propagates the data through two consecutive convolution blocks and one fully connected layer. As in Angermueller et al. (2017), each convolution block consists of a convolutional layer followed by a max pooling layer. The size of filters and their amounts are indicated in Figure 1. Data normalization is known to facilitate the training by both speeding up the training process and making it less sensitive to different choices of hyperparameters, such as learning rate. Thus, following the max pooling layer in each convolution block, we added a batch normalization layer.

A bi-directional gated recurrent unit (GRU) network (Cho et al., 2014) was used to exploit the methylation correlation among neighboring CpGs and learn such correlation from multiple methylome profiles. The input to a GRU is a vector of size of 100, composed of concatenating two vectors. One of them contains methylation level of 50 CpG sites surrounding the one to predict for, 25 on each side. The other vector includes the base pair distance from the corresponding surrounding CpG sites to the one to predict for. The features learned from passing through the sequential input in two opposite directions were combined by simple concatenation to produce the final representation of learned methylation correlation among neighboring CpG sites (Figure 1).

To fuse sequence features and methylation patterns, the Joint subnetwork propagates the combined representation (by concatenation) from Sequence and Methylation subnetworks through two fully connected layers. Like in Sequence subnetwork, batch normalization is used following each fully connected layer to facilitate network training.

With features extracted by the three feature learning subnetworks, the methylation state prediction for a targeted CpG can be made with a Classification subnetwork head. The input to this classification head is determined by the data to consider in the prediction. Specifically, the output of the Sequence subnetwork is used when only surrounding DNA sequence patterns are utilized. Similarly, when only local methylation patterns are considered, the output of the Methylation subnetwork should be used. If to take into account both the sequence and methylation patterns, the output of the Joint subnetwork is used. The classification head includes a fully connected layer followed by a Softmax layer (not shown in Figure 1). Multi-task learning has been widely used to improve model performance in many applications, including prediction for functional genomics events (Zhou and Troyanskaya, 2015; Avsec et al., 2021). In multi-task learning, multiple models are jointly trained with sharing certain components of the models, allowing mutual learning among tasks to improve performance. In this work, we also leverage multi-task learning to jointly train networks for multiple methylome profiles, with predicting for each profile being a separate learning task. All tasks share the same feature extraction subnetworks, but with task-specific classification head as illustrated in Figure 1.

### 3.3 Loss Function

To train DNNs for predicting varying functional genomic events including DNA methylation, the logistic loss has been the primary loss function utilized so far in the literature. Let **y** ∈ {0,1}^*N*^ denote the vector containing true labels and $\tilde{y}\in {\left[0,1\right]}^{N}$ represent the corresponding predicted probabilities, and the logistic loss (*LL*) is calculated as shown below:

$$LL\left(y,\tilde{y}\right)=\overset{N}{\underset{i=1}{\sum }}-y_{i}\log\left({\tilde{y}}_{i}\right)-\left(1-y_{i}\right)\log\left(1-{\tilde{y}}_{i}\right).$$ (1)

In the obtained methylome profiles, the methylation state of a CpG is characterized by the fraction number of reads that contain methylated cytosine out of the total number of reads that overlap with the CpG. In other words, the methylation state of any CpG is a value in [0, 1] and a CpG (*s*_1_) with a value of 0.51 is expected to be in very different methylation state compared to another CpG (*s*_2_) with a value of 0.99. However, to compute the logistic loss as in Eq. 1, the methylation state needs to be converted to a binary value (as *y*_*i*_ ∈ {0, 1}) by comparing to pre-defined threshold, typically 0.5. More specifically, CpGs with an assessed methylation state in a profile above 0.5 would be considered as methylated and labeled with 1 in the profile; while CpGs would be considered as unmethylated and labeled with 0 if their assessed state is below 0.5. Such a conversion results in no difference at all in the methylation state between *s*_1_ and *s*_2_, as both would be labeled with 1 (i.e., methylated). The information loss during this process may lead to suboptimal models.

To make use of the most information carried in the profiles for training, we propose to utilize KL divergence score (*D*_*KL*_) which needs no binary conversion of the methylation state. KL divergence measures the difference between two distributions. In our problem of predicting for the methylation state of a CpG (*i*), the empirically assessed (true) state (*y*_*i*_ ∈ [0, 1]) and the predicted state ( ${\tilde{y}}_{i}\in \left[0,1\right]$ ) can be seen as two distributions. KL divergence, as calculated in Eq. 2, can be used to measure the difference between true and predicted states. The optimization goal here is to find a network producing a prediction that minimizes the $D_{KL}\left(y_{i},{\tilde{y}}_{i}\right)$ .

$$D_{KL}\left(y_{i},{\tilde{y}}_{i}\right)=y_{i}\log\left(\frac{y_{i}}{{\tilde{y}}_{i}}\right)+\left(1-y_{i}\right)\log\left(\frac{1-y_{i}}{1-{\tilde{y}}_{i}}\right).$$ (2)

Let **w** be a vector that contains all learnable parameters in the network and **X** denote the network input. Considering all CpGs for training in a profile with simultaneously learning for multiple (*m*) profiles, the following is the overall loss function to minimize by finding the optimal $\hat{w}$ during the network training.

$$\ell \left(w;X,y^{j:j=1\ldots m}\right)=\overset{m}{\underset{j=1}{\sum }}\alpha_{j}\overset{N_{i}}{\underset{i=1}{\sum }}\beta_{i}^{j}D_{KL}\left(y_{i}^{j},{\tilde{y}}_{i}^{j}\right),$$ (3)

Where **y** ^*j*^ represents the true methylation state of CpGs in *j*-th profile and *N*_*j*_ is the number of CpGs in *j*-th profile for training. There are two sets of hyperparameters involved in this loss function: *α*_*j*_’s and $\beta_{i}^{j}$ ’s. The former balances the contribution of each individual task to the overall loss; while the latter specifies that of each individual CpG in every profile.

### 3.4 Transfer Learning

To obtain models for completing methylome profiles of oocytes and early embryos (target profiles), we started from training feature extraction subnetworks: Sequence, Methylation, and Joint, leveraging profiles of somatic tissues and sperm (Table 1) using multi-task learning as illustrated in Figure 1. The trained subnetworks, referred as source models, were subsequently used as pretrained ones to train networks (target models) for target profiles.

#### 3.4.1 Source Model

To study the contributions of DNA sequence and local methylation patterns to the prediction of methylation, we trained models that uses DNA sequence only, methylation state of neighboring CpGs only, or the combination of the two (full model). In addition, we studied three different ways of training to obtain the best performing full model for transferring. The trained models are summarized in below:

Seq: Model that predicts from DNA sequence only, consisting of the Sequence subnetwork followed by the Classification head. The two subnetworks were trained from scratch with randomly initialized network weights.
Met: Model that predicts from methylation state of neighboring CpGs only, consisting of the Methylation subnetwork followed by the Classification head. Same as in Seq model, the two subnetworks were trained from scratch with random initialization.
The following three are all full models that predict from both DNA sequence and methylation state of neighboring CpGs, consisting of all three feature extraction subnetworks followed by the Classification head. They differ in how the full model was built.
Full1: All four subnetworks were trained from scratch with random initialization.
Full2: The Sequence subnetwork in the Seq model and Methylation subnetwork in the Met model were utilized as pretrained subnetworks. The full model was built by training the Joint subnetwork and the classification head from scratch with the two pretrained subnetworks remaining fixed.
Full3: The full model was built in the same way as for Full2 except that the two pretrained subnetworks were fine-tuned during the training.

The three feature extraction subnetworks from the best performing full model were transferred for subsequent model training to predict DNA methylation in target profiles.

#### 3.4.2 Target Model

Given their distinct nature, there is likely variation among the three feature extraction subnetworks in their contribution to the improvement of target models through transferring. To study such differential impact, we trained models with/without transferring for predicting from DNA sequence only, and methylation state of neighboring CpGs only, and both. The detailed description of the explored settings is provided in below.

##### 3.4.2.1 Predicting From DNA Sequence Only

SeqN: The Sequence subnetwork was trained from scratch (i.e., without transferring) together with the Classification head.
SeqT1: The Sequence subnetwork was initialized using the transferred source model and remained fixed during the training for the Classification head.
SeqT2: The Sequence subnetwork was initialized with the transferred source model and fine-tuned while training for the Classification head.

##### 3.4.2.2 Predicting From Methylation State of Neighboring CpGs Only

MetN: The Methylation subnetwork was trained from scratch together with the Classification head.
MetT1: The Methylation subnetwork was initialized with the transferred source model and remained fixed during the training for the Classification head.
MetT2: The Methylation subnetwork was initialized with the transferred source model and fine-tuned while training for the Classification head.

##### 3.4.2.3 Predicting From Both DNA Sequence and Methylation State of Neighboring CpGs

FullN: All three feature extraction subnetworks were trained without transferring together with the Classification head.
FullTS1: Sequence subnetwork was transferred but remained fixed during the target model training. The other two feature extraction subnetworks were trained without transferring together with the Classification head.
FullTS2: Identical to FullTS1 except that the transferred Sequence subnetwork was fine tuned.
FullTM1: Similar to FullTS1 but with Methylation subnetwork being the only transferred subnetwork.
FullTM2: Identical to FullTM1 except that the transferred Methylation subnetwork was fine tuned.
FullTB1: Both Sequence and Methylation subnetworks were transferred but remained fixed during the target model training. The Joint subnetwork was trained without transferring together with the Classification head.
FullTB2: Identical to FullTB1 except that the two transferred subnetworks were fine tuned.
FullTA1: All three feature extraction subnetworks were transferred but remained fixed during the training for the Classification head.
FullTA2: Identical to FullTA1 except that all transferred subnetworks were fine tuned.

### 3.5 Network Training and Evaluation

The networks were implemented and the experiments were carried out using TensorFlow framework in Python, a popular open-source software library in deep learning research. To train and evaluate all the networks, we partitioned the methylome profile into three parts by chromosomes that were used for training, validation, and testing, respectively. More specifically, data from chromosomes 1, 4, 7, 10, 13, 16, 19, 22, 25, and 28 were used for training to optimize network weights. Data from chromosomes 3, 6, 9, 12, 15, 18, 21, 24, and 27 were used for validation to identify optimal setting for hyperparameters, such as learning rate. Data from chromosomes 2, 5, 8, 11, 14, 17, 20, 23, 26, and 29 were used for testing to evaluate the performance of all trained networks. Adam optimizer (Kingma and Ba, 2015) was used to optimize network weights with weight decay and early stopping. All networks were trained with applying both *ℓ*_1_ and *ℓ*_2_ regularizers and with a mini-batch size of 128. The hyperparameter $\beta_{i}^{j}$ ’s (individual sample weights) in Eq. 3 were specified according to the class label distribution in individual profiles. To simplify, all *α*_*j*_’s (task weights) were set to 1 in this study. For all settings, we fine-tuned the learning rate with grid search from {0.1, 0.01, 0.001, 0.0001, 0.00001, 0.000001}. If not specified otherwise, the performance of the best model among the different choices of learning rate was reported for each setting.

There are different metrics that can be used to evaluate the performance of classification models, such as accuracy, area under curve of receiver operating characteristic (AUC-ROC), area under precision recall curve (AUPRC), and F1 score. Most of existing works on functional genomics events prediction used varying combinations of AUC-ROC, AUPRC, and accuracy Zhang et al. (2015); Angermueller et al. (2017); Liu et al. (2018); Zhang and Hamada (2018). The AUC-ROC and AUPRC take into account the uncertainty in prediction and are not metrics to evaluate the performance of models in making specific binary classification. In addition, these two metrics and accuracy tend to overestimate model performance when there is large imbalance in the class label distribution, which is the case in our study (Table 1). To avoid this problem, we used F1 Score based on the minor class as the primary metric for evaluating models in making specific binary classification.

## 4 Results and Discussion

### 4.1 Comparison of Evaluation Metrics

As indicated in Table 1, the methylation rate, the proportion of methylated CpGs (with a methylation level above 0.5) out of the total in the genome, in target profiles is very low, ranging from 1.97 to 8.39%. This leads to datasets with large imbalance in the class label distribution when labeling methylated CpGs as positive examples and unmethylated as negative ones. In contrast, profiles from GSE106538 and GSE147087 (source profiles) have a methylation rate in a range of 40.81–94.04% with the majority around 80%, which results in a dataset that has much less imbalance in class label distribution. To show the difference among different metrics including AUC (i.e., AUC-ROC), accuracy, and F1 score in cases of large class label imbalance, besides the five models described in the above section (Materials and Methods) trained on source profiles, models using the exact same settings were also trained on target profiles.

**FIGURE 2:**
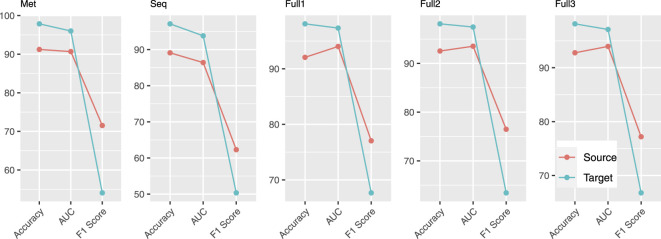
Comparison of accuracy, AUC, and F1 score using models obtained on source and target profiles.

Performance of all models evaluated by three metrics (AUC, accuracy, and F1 score) is provided in Figure 2. According to accuracy and AUC, all five models for target profiles perform better than the corresponding models for source profiles. However, by F1 score the comparison indicates a completely different story, the performance of target models being substantially worse. The reason that accuracy and AUC associated with target profiles are high is the high level of class label imbalance that resulted from extensive low methylation rate. In an extreme case, a classifier that does not learn any intrinsic patterns in the data that are predictive of methylation and simply predicts every example to be negative after just learning the class label distribution can achieve an accuracy above 91%. Therefore, in the presence of large class imbalance, F1 score, specifically the F1-score calculated with labeling the minor class as positive, is a better metric to use for evaluating how well a classifier learning intrinsic patterns from the data.

### 4.2 Models for Source Profiles

To demonstrate the advantage of using KL divergence as the training objective over logistic loss and MSE with/without sigmoid mapping, we trained models in all five settings (see Materials and Methods) using all losses on the source profiles. The performance of obtained models using KL divergence and logistic loss measured by F1 score is provided in Table 2 (Supplementary Table S1 for corresponding results when MSE was used). KL divergence outperforms logistic loss in all settings. Specifically, the average F1 scores of models trained to predict from DNA sequence only, neighboring CpG methylation states only, and both are 0.7124, 0.6035, and 0.7700, respectively with KL divergence compared to 0.6991, 0.5830, and 0.7585 with logistic loss. Models trained with KL divergence also have better performance than those trained using MSE with or without the sigmoid mapping (Supplementary Table S1).

**TABLE 2:** F1 score of models obtained on source profiles. Models trained using both logistic loss and KL divergence as the objective function are included for comparison.

Profile	Logistic loss					KL divergence				
	Met	Seq	Full1	Full2	Full3	Met	Seq	Full1	Full2	Full3
Sperm	0.9560	0.8919	0.9585	0.9557	0.9588	0.9584	0.8964	0.9593	0.9595	0.9593
MamGl	0.8768	0.7965	0.8940	0.8917	0.8952	0.8830	0.8070	0.8986	0.8964	0.8984
PreCor	0.7910	0.6898	0.8130	0.8080	0.8119	0.8010	0.6922	0.8198	0.8156	0.8207
WBC1	0.8603	0.7760	0.8913	0.8851	0.8915	0.8753	0.7786	0.8947	0.8917	0.8934
WBC2	0.7188	0.6006	0.7767	0.7499	0.7740	0.7434	0.5916	0.7867	0.7788	0.7870
Adip1	0.7556	0.6321	0.7864	0.7824	0.7866	0.7683	0.6323	0.7944	0.7896	0.7928
Adip2	0.5783	0.5023	0.7137	0.5595	0.6908	0.6540	0.4588	0.7227	0.7190	0.7341
Muscle	0.7459	0.6206	0.7893	0.7803	0.7873	0.7569	0.6379	0.7982	0.7905	0.7959
Heart1	0.5475	0.2910	0.6145	0.5984	0.6155	0.5426	0.3607	0.6024	0.6114	0.6058
Heart2	0.5650	0.4650	0.6976	0.6147	0.6841	0.6140	0.4636	0.6954	0.6945	0.7099
Lung	0.7641	0.6509	0.8000	0.7927	0.7982	0.7729	0.6653	0.8053	0.8005	0.8039
Spleen	0.7463	0.6623	0.7907	0.7785	0.7891	0.7598	0.6555	0.7928	0.7892	0.7957
Liver1	0.5832	0.4178	0.6490	0.6298	0.6492	0.5674	0.4426	0.6473	0.6393	0.6532
Liver2	0.4855	0.3829	0.6116	0.5319	0.5900	0.5024	0.3968	0.6219	0.6008	0.6252
Ileum	0.6702	0.6022	0.7721	0.7512	0.7654	0.6678	0.6461	0.7813	0.7710	0.7830
Rumen	0.7493	0.6120	0.7963	0.7909	0.7999	0.7586	0.6918	0.8052	0.8005	0.8059
Jejun	0.6173	0.5453	0.7780	0.7221	0.7756	0.6238	0.6180	0.7913	0.7756	0.7841
Kidn1	0.6690	0.5564	0.7584	0.7044	0.7395	0.6999	0.5688	0.7602	0.7581	0.7719
Kidn2	0.5741	0.4029	0.6239	0.6065	0.6223	0.5579	0.4169	0.6246	0.6109	0.6168
Uterus	0.6181	0.4919	0.6775	0.6579	0.6754	0.6291	0.4840	0.6840	0.6806	0.6821
Ovary	0.8088	0.7026	0.8549	0.8463	0.8544	0.8166	0.7459	0.8609	0.8565	0.8610
Placenta	0.7000	0.5323	0.7405	0.7361	0.7329	0.7194	0.6265	0.7523	0.7488	0.7592
Average	0.6991	0.5830	0.7631	0.7352	0.7585	0.7124	0.6035	0.7681	0.7627	0.7700

Among the five models, there are three that were trained to predict methylation from both sequence and neighboring CpGs in different settings (see Materials and Methods). The results in Table 2 indicate fine-tuning the separately trained methylation and sequence subnetworks while training the joint subnetwork (Full3) leads to the full model that has the best average performance. This is consistent between the use of different objective functions, even though with logistic loss Full3 is only minimally better than training all subnetworks from scratch (Full1). The result from comparing Full3 to Full2 suggests fine-tuning the pretrained methylation and sequence subnetworks is necessary to obtain models with better performance. The three subnetworks obtained in Full3 were subsequently used as pretrained networks to obtain models for target profiles.

**FIGURE 3:**
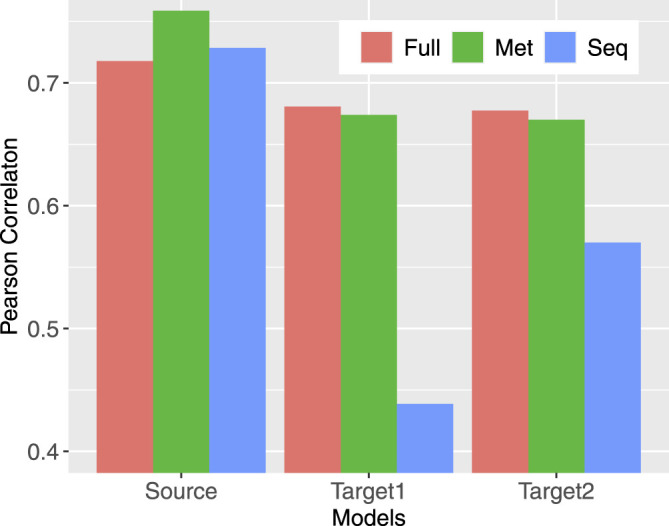
Pearson correlation between model performance and the data coverage rate in corresponding profiles. Source indicates the models trained for source profiles; Target1 (Target2) labels models trained on target profiles without (with) transferring.

Compared models trained to predict methylation from sequence only (Seq), from neighboring CpG methylation states only (Met), and from both (Full), the Full model always has the best prediction performance regardless the objective function being used (Table 2). Models predicting from neighboring CpGs perform much better (average F1 score: 0.7124) than that predicting from sequence (average F1 score: 0.6035). The performance of all three model variants for individual profiles closely correlates with the data coverage rate in corresponding profiles with a Pearson correlation ranging from 0.72 to 0.76 (Figure 3). In other words, the higher the coverage rate is in a profile, the better performance the corresponding models are likely to have. Among the three scenarios, the performance of models predicting from methylation state of neighboring CpGs is the one that mostly correlates the data coverage (*cor* = 0.76). This makes sense because the higher the data coverage rate is, the local CpG methylation pattern is more informative to the prediction for the target CpG.

### 4.3 Models for Target Profiles

To find out how transfer learning helps with obtaining models for target profiles, we trained networks in varying settings (see Materials and Methods) using KL divergence as the objective function. The subnetworks that were transferred are those in Full3 trained for source profiles also with KL divergence as the objective function. The performance of all models assessed using F1 score is provided in Table 3 (Supplementary Table S2 for other performance metrics, including accuracy, AUC-ROC, precision, and recall). Similar to the case with source profiles, models predicting from both sequence and neighboring CpGs perform better than those predicting from anyone of them only with one exception: 4-cell, for which predicting from CpG only (F1 score: 0.8367) is slightly better than predicting from both (F1 score: 0.8357). Models trained with transferring all three subnetworks together with subsequent fine-tuning (FullTA2) achieved the overall best performance across profiles (average F1 score: 0.6828). Aligning with the observations in training for source profiles, the performance of models for individual profiles also well positively correlates with the data coverage rate, but with reduced correlation, especially in the case of predicting from sequence only (Figure 3). Such much-reduced correlation is likely due to the extremely high sparsity in several profiles that leads to overfitting. This is evidenced by the much-improved correlation (from 0.44 to 0.57) when more data were considered via model transferring. For profiles that have extremely low coverage rate, including GVO, MIIO1, and MIIO2, predicting from neighboring CpGs only does not perform well with F1 score ranging from only 0.1754 to 0.2868, much worse than predicting from sequence only.

**TABLE 3:** F1 score of models trained for target profiles in varying transfer settings.

Setting	GVO	MIIO1	MIIO2	2-Cell	4-Cell	8-Cell	16-Cell	Average
SeqN	0.4997	0.4817	0.5214	0.4918	0.5407	0.3546	0.5564	0.4923
SeqT1	0.0815	0.0074	0.0410	0.0071	0.5500	0	0.3749	0.0810
SeqT2	0.4756	0.4553	0.5045	0.4968	0.5003	0.3347	0.5643	0.4759
MetN	0.1338	0.1070	0.1807	0.7432	0.8126	0.4369	0.8004	0.4592
MetT1	0.2625	0.1332	0.1989	0.7634	0.8204	0.4924	0.7995	0.4958
MetT2	0.2868	0.1754	0.2211	0.7435	0.8367	0.5036	0.8062	0.5104
FullN	0.5101	0.5107	0.5668	0.7649	0.7955	0.5165	0.7950	0.6372
FullTS1	0.2922	0.1108	0.2905	0.7334	0.8180	0.5086	0.7809	0.5049
FullTS2	0.5791	0.5483	0.5987	0.7177	0.7791	0.5540	0.7690	0.6494
FullTB1	0.3641	0.2954	0.2244	0.7463	0.8037	0.4944	0.8118	0.5343
FullTB2	0.5733	0.5316	0.5858	0.7806	0.8085	0.5933	0.8143	0.6696
FullTA1	0.4094	0.3345	0.4017	0.7364	0.8004	0.4945	0.8027	0.5685
FullTA2	0.6275	0.5279	0.6380	0.7770	0.8357	0.5630	0.8102	0.6828

Model transferring helped to obtain models with significantly improved performance to predict from neighboring CpGs only or from both sequence and neighboring CpGs. However, there is no gain to be seen in training models predicting from sequence only, except for profiles from 2-cell and 16-cell stages that have the highest data coverage rate among all target profiles. Such lack of improvement is likely due to the extremely low data coverage, causing the learning to arrive in a local minimal that is difficult to reach when training starting from a pretrained sequence subnetwork. The results in Table 3 also indicate that fine-tuning the transferred models always helped, with just very few exceptions. In the case of predicting from sequence only, without fine-tuning, the obtained models almost completely failed to perform for all profiles except those at 4-cell and 16-cell stages, which have relatively higher data coverage rate (0.21 and 1.47%, respectively). Fine-tuning has the least impact on training models to predict from neighboring CpGs only, which suggests that the methylation subnetwork trained using one dataset is ready for using in models for another dataset.

To differentiate the impact of model transferring and KL divergence on models for target profiles, we trained models predicting from both sequence and neighboring CpGs in two additional settings that are using logistic loss as the training objective with and without model transferring. The performance (in F1 score) of these models, together with those trained using KL divergence with/without transferring, is presented in Table 4 (Supplementary Table S3 for performance by other metrics). The results indicate that both transferring and the use of KL divergence helped to improve the performance, importantly in distinct ways that are complementary to each other, since the combination of the two leads to the best-performing models. The improvement from using KL divergence by 29.43% in average F1 score (from 0.4923 to 0.6372) is similar to that from model transferring and much more significant than the similar improvement seen in the model training for source profiles. This again indicates that KL divergence is a more effective objective function to use when training models for DNA methylation prediction. It is also worth noting that both the use of KL divergence and model transferring lead to reduced variance in the performance across profiles (Table 4), with higher reduction seen with transferring. This suggests that the initial worse performing models gained more improvement when leveraging either KL divergence or model transferring.

**TABLE 4:** F1 score of models trained for target profiles using different objective functions and with/without transfer.

Profile	Baseline	KLD	TLR	KLD + TLR
GVO	0.3073	0.5101	0.5840	0.6275
MIIO1	0.5169	0.5107	0.5299	0.5279
MIIO2	0.3750	0.5668	0.5635	0.6380
2-Cell	0.7473	0.7650	0.7311	0.7770
4-Cell	0.8154	0.7955	0.8148	0.8357
8-Cell	0.5169	0.5165	0.4437	0.5630
16-Cell	0.7095	0.7950	0.8124	0.8102
Average	0.4923	0.6372	0.6399	0.6828
SD	0.1931	0.1401	0.1461	0.1237

Baseline: models trained using logistic loss without transfer, corresponding to DeepCpG (Angermueller et al., 2017) with the exception of the addition of batch normalization layers to facilitate training; KLD: KL divergence, models trained using KL divergence without transfer; Trn: transfer, models trained using logistic loss with transfer; KLD + Trn: models trained using KL divergence with transfer; SD: standard deviation.

### 4.4 Imputation for Methylome Profiles of Oocytes and Early-Stage Embryos

**FIGURE 4:**
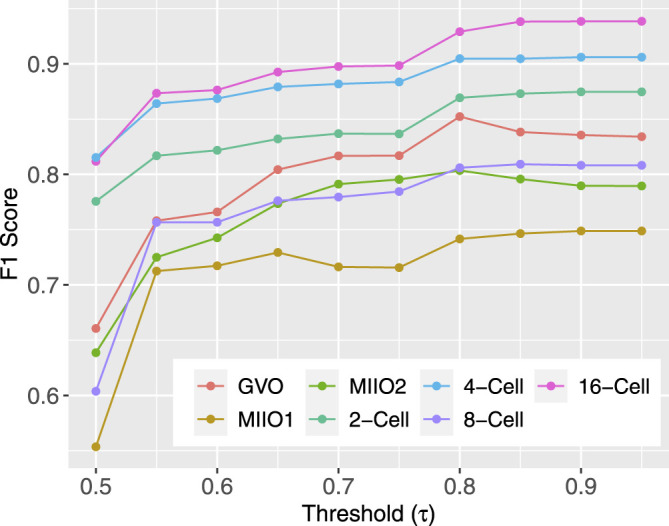
F1 score from the use of varying threshold *τ*.

The best-performing full models on target profiles, that is, those obtained with setting FullTA2 (Table 2) were used to complete the target profiles by imputing the methylation state for CpG sites that do not have experimental data. The models output probabilities of a CpG being methylated in individual profiles. To have the highest possible quality, we used a threshold *τ* and only kept imputed results for CpGs with a predicted probability either above *τ* or below 1 − *τ*. The test data used before for evaluating model performance were leveraged to find the best *τ* to use. For a given *τ*, there was no prediction being made for CpGs in the test set with a predicted probability in between 1 − *τ* and *τ*. These CpGs were not considered in the subsequent F1 score calculation, leading to variation in the F1 score among different choices of *τ*. Intuitively, higher the *τ* is, more certain the prediction is and higher the calculated F1 score is. Figure 4 shows how F1 score varies along with different choices of *τ*, indicating that the improvement in the F1 score becomes minimal for all profiles starting *τ* = 0.8. As a result, 0.8 was used as the threshold in subsequent imputation for CpG sites with missing data.

**FIGURE 5:**
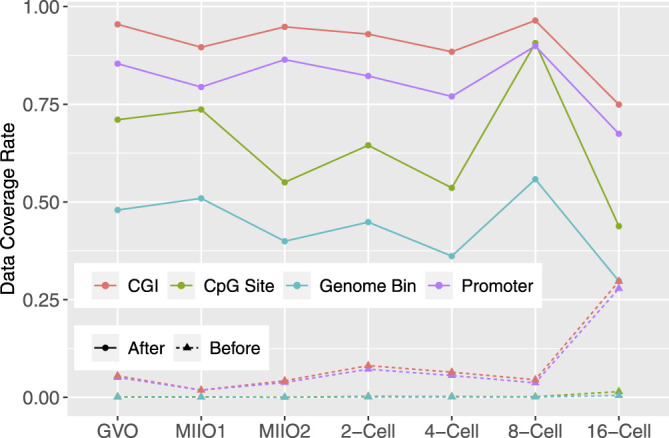
Before and after imputation, the data coverage rate for all CpGs in the genome and three categories of genomic features: promoter, CGI, and 300bp genome bin in methylome profiles of bovine oocytes and early-stage embryos.

Large number of missing CpGs had imputed data in each individual profile, leading to a drastic increase in the data coverage rate from the initial range of 0.06–1.47% to that of 43.80–73.65% (Figure 5). To demonstrate the impact of imputed data on subsequent analyses of functional genomics, we compared the number of genomic features that are considered to have data before and after the imputation. Three categories of genomic features were considered: genome bin, promoter of gene, and CGI. Genome bins were obtained by tiling the reference genome to produce equal-sized and nonoverlapping bins of 300 bp long each. Promoters were defined by 1001bp regions centered at annotated transcription starting site of genes, which were obtained from Ensembl Genome Browser. The CGI annotations were downloaded from UCSC Genome Browser. A genome bin was considered to have data when there were at least three CpG sites with known methylation state within the bin; while given its longer length, a promoter (or a CGI) was considered to have data when there were at least 10 CpG sites with known state within the promoter (or CGI) region. The percentages of genome bins, promoters, and CGIs that were considered to have data out of total 8,869,705, 22,118, and 37,226, respectively, before and after imputation in individual profiles are shown in Figure 5. As for individual CpG sites, substantial increase in the data coverage can be seen for all three categories of genomic features. Specifically, the coverage rate was increased to 29.74–55.80% from 0.02 to 0.48% for genome bins, to 67.44–89.90% from 1.85 to 27.86% for promoters, and to 74.92–96.42% from 1.87 to 29.70% for CGIs. The expanded data will greatly enhance the analyses to understand the mechanisms underlying DNA methylation and its role in regulating various biological functions.

**FIGURE 6:**
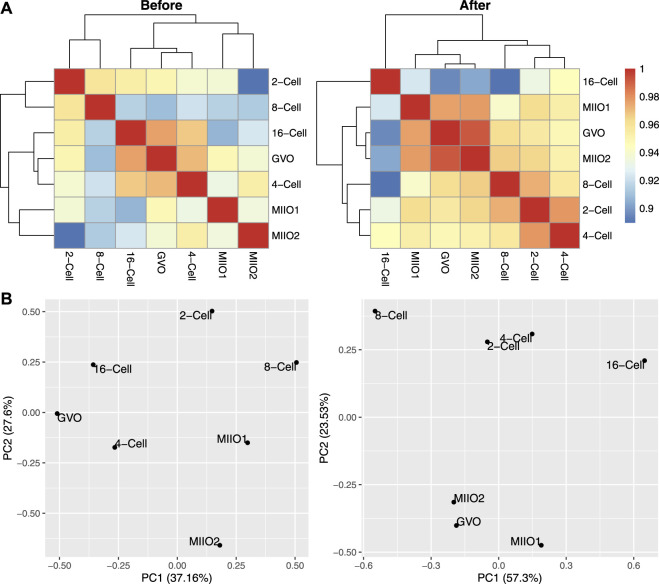
Comparison of DNA methylation profiles of bovine oocytes and early-stage embryos before and after imputation. **(A)**: Pearson correlations among profiles; **(B)**: profiles embedded in the space spanned by the first two principal components.

To demonstrate the impact of imputation on downstream analyses, we calculated the Pearson correlation between each pair of profiles before and after imputation, followed by hierarchical clustering to group profiles. In addition, we performed principal component analysis (PCA) on profiles before and after imputation. The methylation level of 300 bp genome bins (assessed by the average methylation level of CpGs within each bin) was used as input data for these analyses with excluding bins that have missing data in any of the profiles. The results are provided in Figure 6, indicating that imputation helped to obtain profile groupings that better align with existing biological understandings. Specifically, the grouping of the three oocytes profiles and that of 2-cell and 4-cell profiles followed by grouping with 8-cell and 16-cell profiles after imputation (top right, Figure 6) align well with the natural reproductive phases. In contrast, the groupings obtained before imputation (top left, Figure 6) lack clear biological interpretation. The PCA plots with profiles embedded in the two-dimensional space spanned by the first two principal components (bottom panel, Figure 6) also indicate the same story.

## 5 Conclusion

Here, we reported our exploration of utilizing transfer learning and KL divergence in training DNNs to impute for DNA methylome profiles with very low data coverage. The target profiles to complete in our study are those of bovine oocytes and early embryos by WGBS with a data coverage rate ranging from 0.06 to 1.47% after cleaning. To obtain pre-trained models for transferring, WGBS profiles of sperm and a wide range of somatic tissues (coverage rate: 0.85–21.34%) were utilized. The results of our analyses indicate that both model transferring and KL divergence improve the prediction performance of the target models.

Our study demonstrated that KL divergence is a more effective objective function to use than the commonly used logistic loss for training models to prediction DNA methylation. Compared to logistic loss, the use of KL divergence led to models with improved performance in the training for both source and target profiles. Note that KL divergence helps to boost the average F1 score to 0.6372 from 0.4923 across target profiles, which is a much larger increase compared to that seen in source model training (from 0.7585 to 0.7700). This suggests that the use of KL divergence is especially beneficial when the data coverage rate is low, which makes sense as the ability of utilizing as much information carried in the data as possible is of greater importance in the case of limited training set size. Our results also demonstrated that the transferring of models built for profiles with relatively high coverage greatly improves training for those that are in low coverage, with increased average F1 score 0.6399 (from 0.4923). Importantly, model transferring and KL divergence enhance the training of target models in two distinctive ways that are additive, evidenced by the further improved performance (average F1 score: 0.6828) when both were exploited simultaneously. Moreover, our exploration further into the different components of the adopted DNN indicates that local methylation patterns are more transferable across datasets than learned DNA sequence patterns. Finally, to obtain the best models for target profiles, fine-tuning is necessary regardless of which components of the source model are transferred.

The results from the subsequent application of trained models for imputation demonstrated the high effectiveness of our approach in completing DNA methylome profiles that have very low data coverage. Drastic increase in data coverage rate after imputation were seen at both individual CpG sites and varying genomic features, including genome bins, gene promoters, and CGIs. The imputed data would greatly strengthen analyses toward the understanding of biological mechanisms and functional roles of DNA methylation. One of our future works will be to link the methylation level of genomic features to transcriptomic profiles to understand how DNA methylation regulates gene expression as a *cis* regulator. The results from such an analysis will allow more accurate reconstruction of gene regulatory networks underlying a biological system, which is also our future work.

## Data Availability

Publicly available datasets were analyzed in this study. These data can be found at: NCBI GEO: GSE106538, GSE147087, GSE121758. The code, including the implementation of network architecture and the training and evaluating the models is available at the following URL: https://github.com/ODU-CSM/Pub-Met-TL.
