# Supplementary material for: Completing Single-Cell DNA Methylome Profiles via Transfer Learning Together With KL-Divergence
Source: Front Genet. 2022 Jul 22;13:910439. doi: 10.3389/fgene.2022.910439 (PMC9353187; doi:10.3389/fgene.2022.910439)
Supplement: Supplementary file 2 [file DataSheet1.PDF]

Supplementary Material

1 SUPPLEMENTARY TABLES AND FIGURES

Table S1. F1 score of models obtained on source profiles using MSE with/without sigmoid mapping.

Profile	With Sigmoid					Without Sigmoid				
	Met	Seq	Full1	Full2	Full3	Met	Seq	Full1	Full2	Full3
Sperm	0.9579	0.8668	0.9588	0.9591	0.9591	0.9580	0.8727	0.9593	0.9588	0.9592
MamGl	0.8817	0.8068	0.8970	0.8947	0.8961	0.8819	0.7960	0.8972	0.8919	0.8963
PreCor	0.7980	0.6951	0.8201	0.8168	0.8197	0.7954	0.6812	0.8158	0.8083	0.8132
WBC1	0.8738	0.7749	0.8920	0.8894	0.8927	0.8727	0.7641	0.8930	0.8846	0.8929
WBC2	0.7439	0.5949	0.7830	0.7724	0.7826	0.7343	0.5678	0.7809	0.7607	0.7795
Adip1	0.7662	0.6365	0.7929	0.7889	0.7907	0.7660	0.6215	0.7912	0.7822	0.7892
Adip2	0.6445	0.4755	0.7284	0.7206	0.7174	0.6457	0.3975	0.7282	0.7076	0.7127
Muscle	0.7546	0.6333	0.7950	0.7892	0.7917	0.7538	0.6054	0.7948	0.7793	0.7907
Heart1	0.5205	0.3655	0.6177	0.6012	0.6226	0.5299	0.2939	0.6084	0.5784	0.6070
Heart2	0.6000	0.4639	0.7063	0.6895	0.7051	0.6057	0.3958	0.7106	0.6861	0.7057
Lung	0.7712	0.6686	0.8037	0.8005	0.8029	0.7690	0.6477	0.8035	0.7938	0.8020
Spleen	0.7559	0.6574	0.7939	0.7879	0.7930	0.7547	0.6448	0.7941	0.7791	0.7922
Liver1	0.5660	0.4287	0.6552	0.6458	0.6488	0.5701	0.3884	0.6443	0.6140	0.6443
Liver2	0.5089	0.3993	0.6285	0.6089	0.6185	0.4994	0.3275	0.61154	0.5776	0.6183
Ileum	0.6604	0.6417	0.7817	0.7668	0.7791	0.6555	0.5702	0.7799	0.7382	0.7730
Rumen	0.7573	0.6941	0.8044	0.7994	0.8043	0.7566	0.6545	0.8062	0.7941	0.8037
Jejun	0.6185	0.6200	0.7887	0.7708	0.7867	0.6271	0.5135	0.7893	0.7474	0.7849
Kidn1	0.6976	0.5763	0.7633	0.7579	0.7409	0.6954	0.5212	0.7611	0.7489	0.7573
Kidn2	0.5635	0.4162	0.6209	0.6146	0.6231	0.5573	0.3829	0.6181	0.5898	0.6117
Uterus	0.6203	0.4685	0.6865	0.6803	0.6807	0.6193	0.43817	0.6854	0.6572	0.6762
Ovary	0.8162	0.7401	0.8596	0.8548	0.8567	0.8169	0.7062	0.8605	0.8462	0.8592
Placenta	0.7136	0.6226	0.7557	0.7515	0.7558	0.7219	0.6135	0.7584	0.7472	0.7532
Average	0.7087	0.6021	0.7697	0.7619	0.7667	0.7085	0.5638	0.7678	0.7487	0.7487
